# Predictive value of Cardiac Magnetic Resonance: new and old parameters in the natural history of repaired Tetralogy of Fallot

**DOI:** 10.1186/s12872-023-03671-4

**Published:** 2024-01-03

**Authors:** Paola Franceschi, A. Balducci, E. Nardi, F. Niro, D. Attinà, V. Russo, A. Donti, E. Angeli, G. D. Gargiulo, L. Lovato

**Affiliations:** 1 Pediatric and Adult CardioThoracic and Vascular, Oncohematologic and Emergency Radiology Unit, IRCCS Azienda Ospedaliero-Universitaria di Bologna, Via G. Massarenti, 9, Bologna, 40138 Italy; 2 Pediatric Cardiology, Pediatric Cardiac Surgery and Adult Congenital Heart Disease Program, Department of Cardio-Thoracic and Vascular Medicine, IRCCS Azienda Ospedaliero- Universitaria di Bologna, Via G. Massarenti, 9, Bologna, 40138 Italy; 3 Cardiology Unit, Cardiac Thoracic and Vascular Department, IRCCS Azienda Ospedaliero- Universitaria di Bologna, Via G. Massarenti, 9, Bologna, 40138 Italy; 4 Department of Surgical and Medical Sciences, University of Bologna, Bologna, Italy

**Keywords:** Cardiac Magnetic Resonance, Tetralogy of Fallot, Congenital Heart Disease, Pulmonary valve replacement, Homograft

## Abstract

**Background:**

Patients with repaired Tetralogy of Fallot (rTOF) often develop pulmonary regurgitation (PR) and right ventricle (RV) dysfunction, experiencing increased mortality and morbidity rates in adulthood. Pulmonary valve replacement (PVR) timing to address PR is controversial. Cardiac Magnetic Resonance (CMR) is the gold standard for morpho-functional evaluation of complex cardiopathies. This study aims to identify CMR parameters predictive of adverse outcomes to help defining the best therapeutic management of rTOF patients.

**Methods:**

130 rTOF patients who underwent CMR (2006–2019) were enrolled in this retrospective single-center study. CMR, clinical, ECG and exercise data were analyzed. Univariate and multivariate analyses identified clinical and CMR parameters predictive of adverse outcomes both individually (e.g., death, arrhythmias, heart failure (HF), pharmacological therapy, QRS ≥ 160ms) and as composite outcome.

**Results:**

Univariate analysis confirmed RV volumes and RV ejection fraction corrected for PR as adverse outcome predictors and identified interesting correlations: pulmonary artery bifurcation geometry and abnormal interventricular septum (IVS) motion with arrhythmias (p < .001; p = .037), HF (p = .049; p = .005), composite outcome (p = .039; p = .009); right atrium (RA) dimensions with the composite outcome and the outcomes individually (p < .001). The best predictive models by multivariate analysis included sex (male), RV and RA dilation for QRS ≥ 160ms, time form repair to CMR, age at TOF repair and IVS fibrosis for pharmacological therapy.

**Conclusions:**

Besides RV volumes, new adverse prognostic factors could guide rTOF therapeutic management: pulmonary arteries morphology, abnormal IVS motion, RV dysfunction, RA dilation. Perspective multicentric evaluation is needed to specify their effective role.

**Supplementary Information:**

The online version contains supplementary material available at 10.1186/s12872-023-03671-4.

## Background

Tetralogy of Fallot (TOF) surgical repair is usually performed in the early years of life, sometimes preceded by palliative shunt procedures. Most patients with repaired TOF (rTOF) reach adulthood (20-year survival rates exceeding 90%), but often develop hemodynamically relevant pulmonary regurgitation (PR). PR leads to a complex cascade of pathophysiologic events resulting in right ventricle (RV) dilation and dysfunction, left ventricular (LV) dysfunction, exercise intolerance, arrhythmia, and premature death. Thus, mortality and morbidity rates increase substantially from the third decade of life [1, 2]. Pulmonary valve replacement (PVR) is increasingly performed in patients with rTOF to restore valve function and to halt or reverse the adverse ventricular remodeling, but PVR timing is controversial [3, 4]. Cardiovascular magnetic resonance (CMR) is the gold standard for noninvasive assessment of biventricular size and function and quantification of valvular regurgitation. Therefore, it is fundamental for monitoring Adult Congenital Heart Disease (ACHD) especially rTOF. Guidelines for PVR timing rely on upper threshold values for RV volumes below which PVR intervention is recommended, but these values represent markers of suboptimal postoperative ventricular remodeling rather than adverse clinical outcomes [5, 6]. The use of these criteria for reintervention may not correlate with clinical benefit and improvement in survival still needs to be shown. Other cardiac morpho-functional parameters, such as RV mass, have been suggested as potential prognostic factors, but not focused on modifying surgical PVR timing and with relatively limited case series. The goal of this study is to identify new CMR morpho-functional parameters that represent risk factors for adverse outcomes and help to define the best therapeutic management in a large cohort of patients with rTOF.

## Methods

### Patient selection

Retrospective observational study with Institutional Ethical Board approval (Comitato Etico-Area Vasta Emilia Centro (CE-AVEC) code: 22/2019/Oss/AOUBo; protocol: RMC4TDF). Written informed consent was obtained from adult patients and from a parent and/or legal guardian for minor patients. Patients followed on an outpatient basis by the Adult Congenital Heart Disease Program, Department of Cardio-Thoracic and Vascular Medicine, IRCCS Azienda Ospedaliero-Universitaria di Bologna were consecutively enrolled in this single-center study. Patients fulfilling the following criteria were included: [1] repaired TOF (non-conduit and without pulmonary valve reconstruction or implantation); [2] at least one CMR completed between 1st January 2006 to 31st March 2019 as part of the normal care pathway; [3] age over 16 at the time of the first CMR; [4] clinical follow-up ≥ 2 years. The clinical follow-up period of each patient extends from the first CMR until 31st March 2021. Patients with incomplete CMR imaging data or not suitable for quantitative assessment were excluded. CMR, clinical, ECG and exercise testing data refer to the first CMR performed within the observation period and the closest cardiological visit. In patients who underwent at least two CMRs during the observation period before eventual PVR, two CMRs were analyzed for the evaluation of ventricular volume increase over time. Outcomes’ data cover the entire observation period. Data were obtained by reviewing both paper and digitized medical records.

### CMR imaging

Studies were performed using Signa 1.5 Tesla (GE, Milwuakee, Wisconsin) scanner from the 1st January 2006 to the 31st May 2016, and Ingenia 1.5 Tesla (Philips, Rotterdam, The Netherlands) scanner from the 1st June 2016 to the 31st March 2019. The post-processing analysis was performed using Circle Cardiovascular Imaging software (Circle CVi^42^, Calgary, Canada). Measurements were conducted by investigators who were blinded to patient clinical outcomes. Ventricular volumes, mass and function were measured on cine balanced steady-state free precession (bSSFP) two-chamber short-axis images using four-chamber and two-chamber long-axis images as cross-reference. LV and RV mass-to-volume ratios were calculated dividing the ventricular mass by the EDV. These parameters were evaluated as continuous variables. Some of them were also evaluated as categorical variables and the predictive value for adverse events of proposed thresholds was assessed. Pulmonary regurgitant volume and fraction were calculated on Phase Contrast (PC) images passing through the pulmonary valve. RVEF corrected for pulmonary regurgitation (RVcEF) was calculated as:

$$RVcEF=\frac{netPFV}{RVEDV}\cdot 100=RVEF\cdot \left(1-PRF\right)\cdot 100$$

**Fig. 1:**
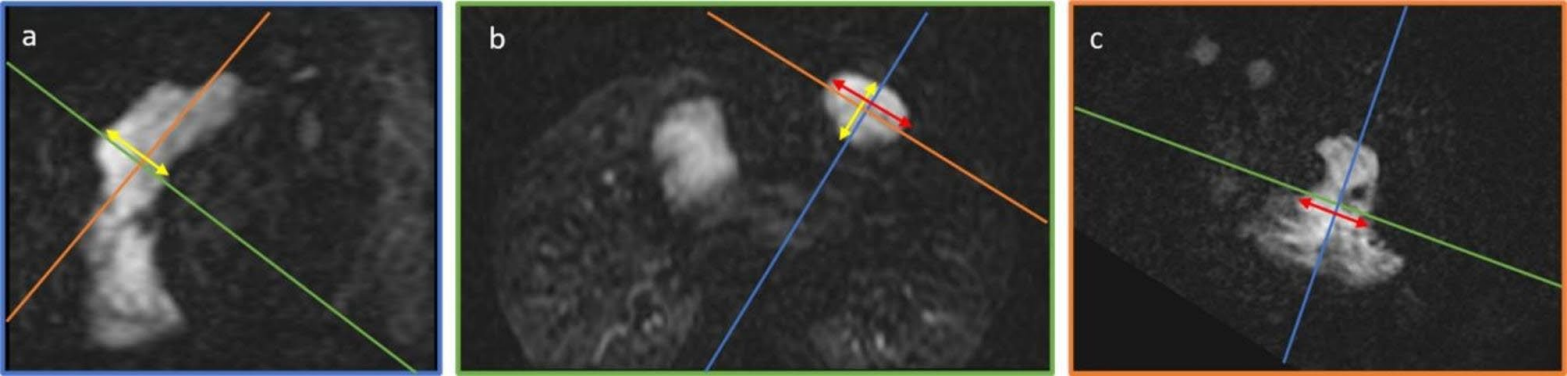
Measurement of RVOT aneurysm coronal and sagittal diameters on MIP reconstructions of ceMRA images. LEGEND: Sagittal (**a**, blue frame), transverse (**b**, green frame) and coronal (**c**, orange frame) RVOT sections. The greatest sagittal and coronal RVOT aneurysm diameters are respectively displayed as yellow (**a-b**, sagittal) and red (**b-c**, coronal) double arrowed segments. RVOT = right ventricular outflow tract; MIP = maximum intensity projection; ceMRA = contrast enhanced MR angiography

Where netPFV is net Pulmonary Forward Volume and PRF is Pulmonary Regurgitant Fraction [7]. The maximum diameters (latero-lateral and apicobasal) and area of the right atrium (RA) were manually traced on the ventricular 4-chamber view in atrial diastole (ventricular systole) with care taken to avoid measurement of the cavae entering the RA. Tricuspid annular plane systolic excursion (TAPSE) was measured on a middle ventricular 4-chamber view bSSFP cine image as the distance between the cutting edge of tricuspid annulus with RV free wall at end-diastole and at end-systole. Myocardial fibrosis detected by late Gadolinium enhancement (LGE) on RV anterior wall and IVS was evaluated on Delayed Enhancement (IR-FGRE T1) 2 chambers images both visually as categorical variable (present/absent) and as continuous variable (extent) on the slice where it was most represented. RVOT aneurysm presence/absence was established on bSSFP right ventricular outflow tract (RVOT) cine images and defined as outward movement during systole of part of the ventricular wall or its reconstructed outflow tract [8]. RVOT aneurysm coronal and sagittal diameters were measured on maximum intensity projection (MIP) reconstructions of contrast enhanced MR angiographic (ceMRA) images on corresponding planes (Fig. 1).

**Fig. 2:**
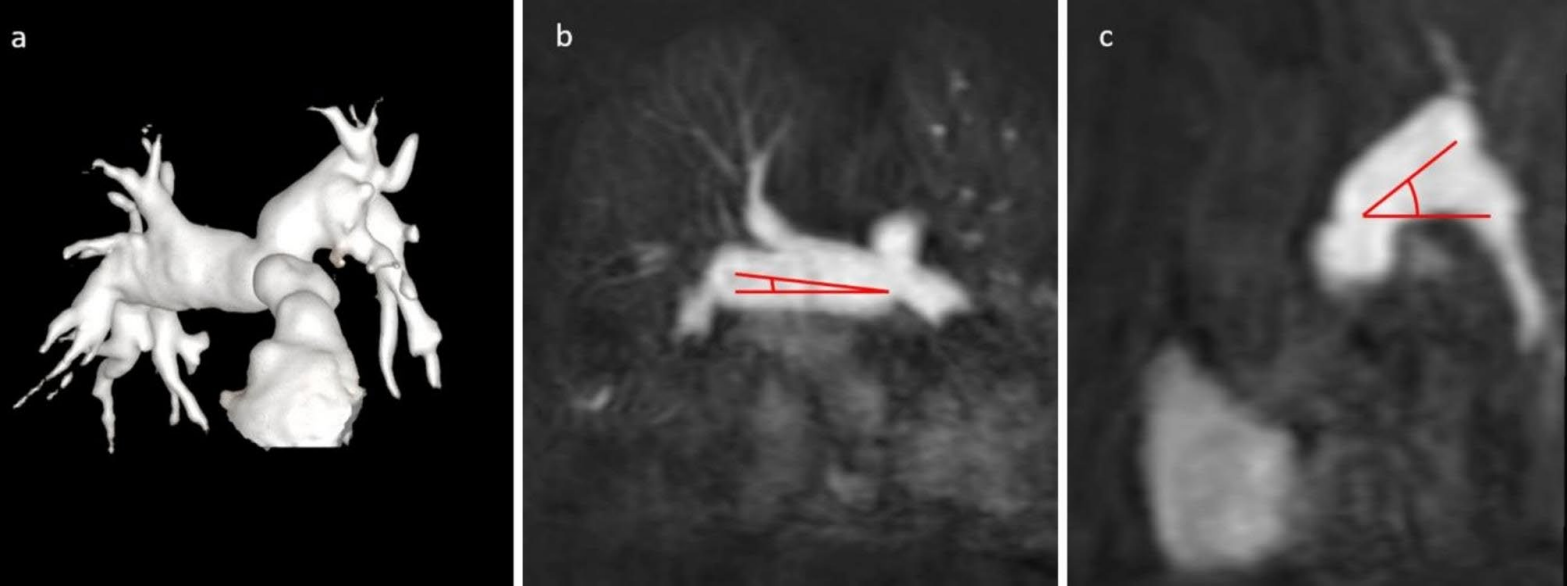
Main pulmonary artery bifurcation geometry with steep angulation of the LPA. LEGEND: The figure shows an anterior view of the main pulmonary artery with steep angulation of the LPA. The patient developed sustained VT and heart failure, which required hospitalization. Anterior view of a 3D volume rendered image (**a**); right anterior oblique view (**b**) and left anterior oblique view (**c**) of ceMRA MIP reconstructions with cranio-caudal angulation of RPA (**b**) and LPA (**c**). MIP = maximum intensity projection; ceMRA = contrast enhanced MR angiography; RPA = right pulmonary artery; LPA = left pulmonary artery

RVOT-MPA morphology was visually evaluated on 3D ceMRA reconstructions and classified into 6 distinct shapes (simple tubular, tubular dilated, hourglass, pyramid, funnel and convex) [9, 10]. MPA, RPA and LPA diameters and angles describing the geometry of MPA bifurcation were measured on ceMRA MIP reconstructions according to Knobel et al. [11] (Fig. 2). PAs’ stenosis and hypoplasia were defined by vessel diameter z-score<-2 at a localized point of the vessel or extended to a longer segment of the vessel, respectively. PAs dilation was defined by vessel diameter z-score > 2.

**Fig. 3:**
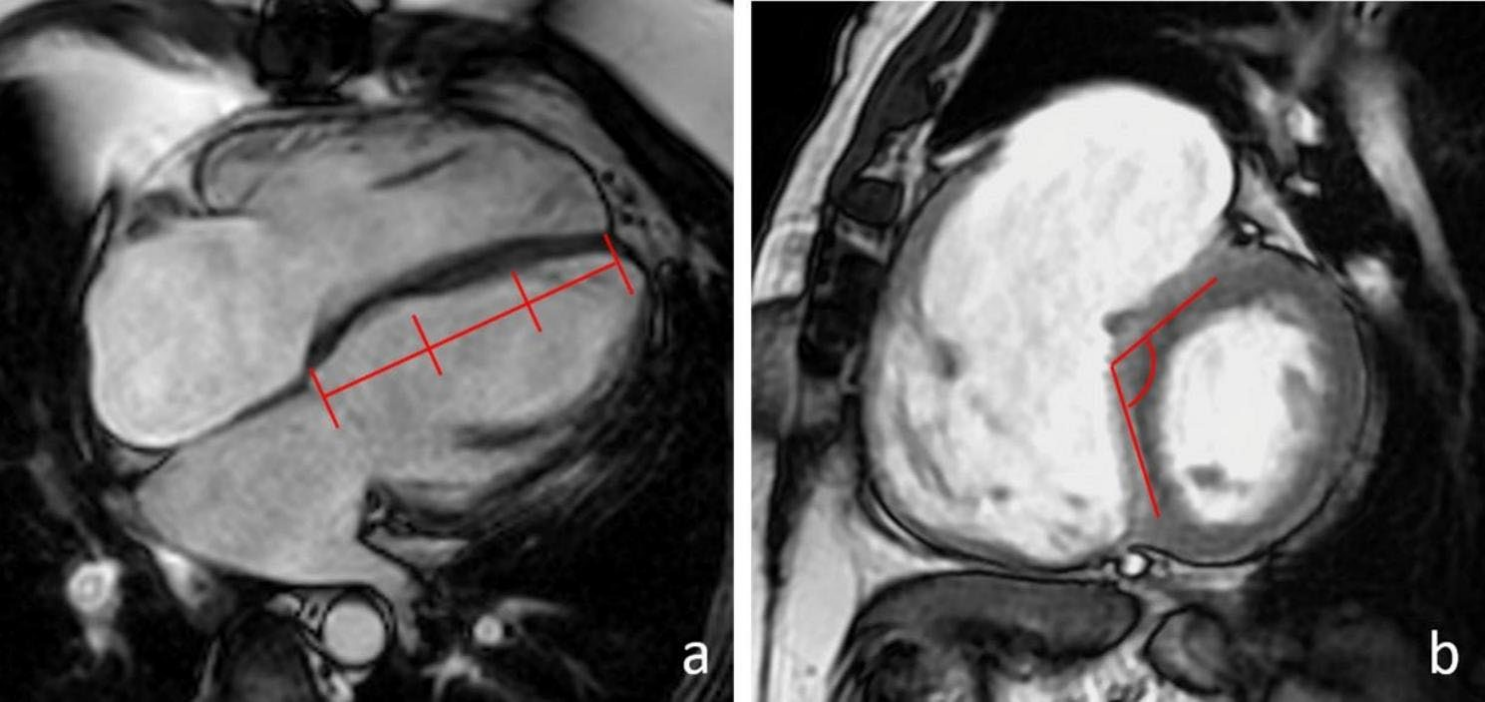
Measurement of abnormal IVS motion extent and IVS flattening angle. LEGEND: Measurement of abnormal IVS motion extent on a 4ch bSSFP cine image (**a**). IVS flattening angle measurement on a 2ch bSSFP cine image (**b**). IVS = interventricular septum; bSSFP = balanced steady-state free precession

**Fig. 4:**
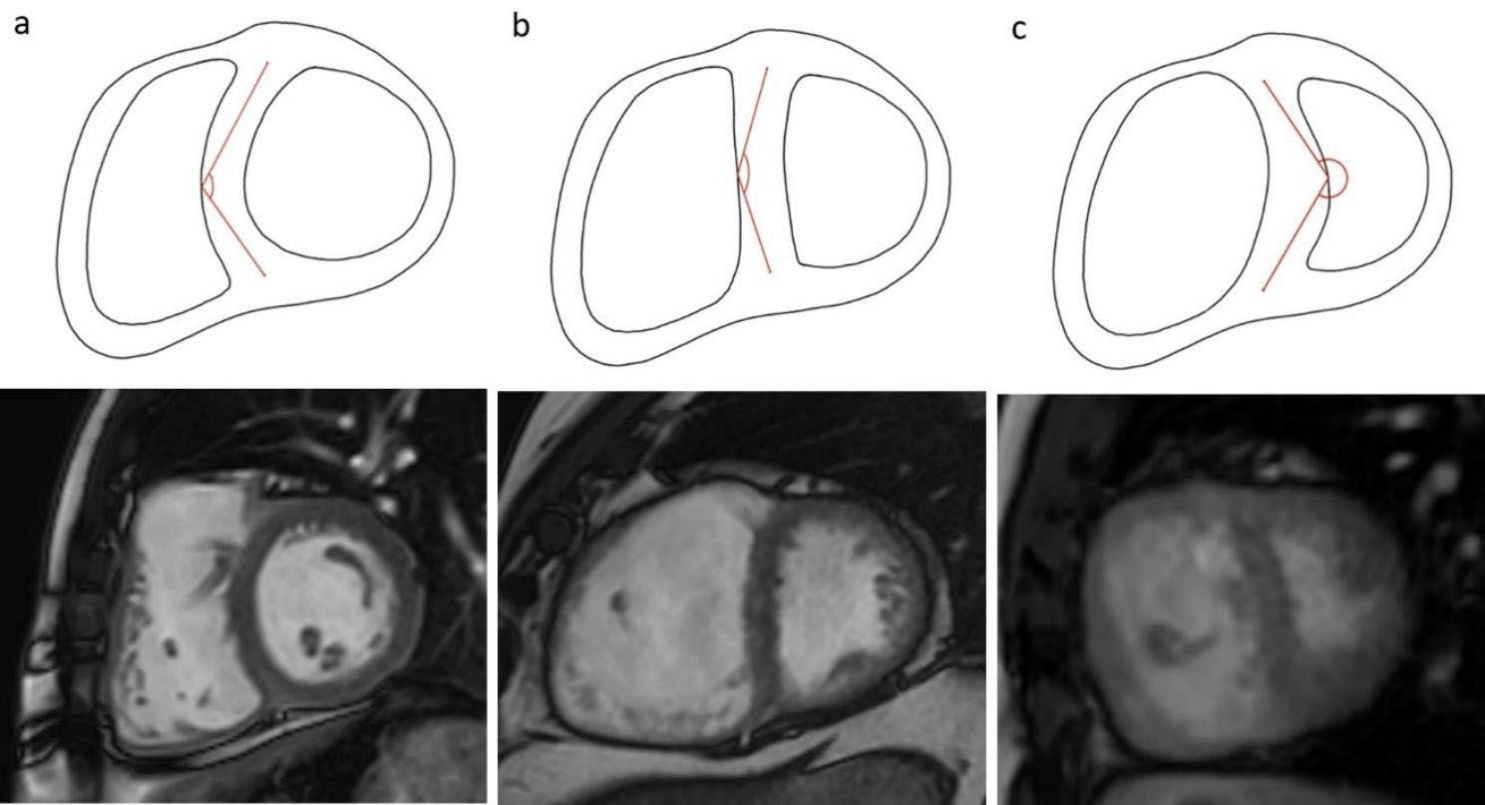
Interventricular septum motion. LEGEND: Schematic representation of IVS flattening angle measurement and corresponding 2ch bSSFP cine images. Normal IVS motion (**a**); IVS flattening (**b**); IVS inversion (**c**). IVS = interventricular septum; bSSFP = balanced steady-state free precession

Type of IVS motion was defined on two-chambers short axis and four-chambers long axis cine bSSFP images as normal or abnormal (diastolic IVS flattening, systo-diastolic IVS flattening or diastolic IVS inversion). Abnormal IVS motion duration was assessed on two-chambers short axis cine bSSFP images (none, protodiastolic, holodiastolic). Abnormal IVS motion extent was identified on four-chambers long axis cine bSSFP images as the IVS segment affected by the abnormal motion (basal or basal-middle, basal-middle-apical) (Fig. 3a). IVS flattening angle was measured on two-chamber short-axis cine bSSFP images at two different time instants of the cardiac cycle specifically in end-diastole and at the time when it was maximal (usually corresponding to proto-diastole). The angle was drawn between the midpoints of the anterior and inferior interventricular insertion points, and the point of the IVS wall most protruding into the RV cavity in normal IVS motion and IVS flattening, and LV cavity in the case of IVS inversion. The measured angle is the one “open” towards the left ventricle, being > 180° in IVS inversion (Figs. 3b and 4).

### Outcomes

The composite outcome included: death, major arrhythmias (paroxysmal supraventricular tachycardia (SVT), persistent SVT, sustained ventricular tachycardia (VT), nonsustained VT, ventricular fibrillation, atrial fibrillation, atrial flutter), heart failure (HF) hospitalization, need for pharmacological therapy, NYHA class increase, QRS ≥ 160ms, VO2max reduction ≥ 5%, RVEDVi increase > 20ml/m^2^, RVESVi increase > 20ml/m^2^. Pharmacological therapy refers to heart failure and antiarrhythmic drug therapy.

### Statistical analysis

CMR, clinical, ECG and exercise data were summarized for all eligible patients using frequencies and percentages for categorical variables and median with interquartile range for continuous variables. Categorical and continuous variables were compared between patients who experienced the composite outcome and those who did not using respectively the chi-squared test and the Wilcoxon rank-sum test as appropriate. The same analyses were repeated comparing the characteristics of patients who experienced the single outcomes separately and those who did not. Univariate and multivariate logistic regression analyses were performed to evaluate risk factors of all the outcomes. Multivariate models building followed a backward-stepwise approach; the test of term significance is the Wald chi-square test with cutoff *p* value of 0.1 for removal and 0.05 for addition. For each multivariate logistic regression, the model discrimination and calibration were reported. Model discrimination was assessed calculating the Area under the Receiver Operator Characteristic (ROC) curve, whereas model calibration has been determined by Hosmer-Lemeshow (H-L) technique. In H-L test, a *p* value higher than 0.05 suggests that observed and predicted probabilities match: the higher the *p* value, the better the model calibration. A two-sided *p* value of less than 0.05 was considered to indicate statistical significance. Statistical analyses were performed using Stata V.17 (StataCorp, College Station, Texas, USA).

## Results

### Study patients

**Fig. 5:**
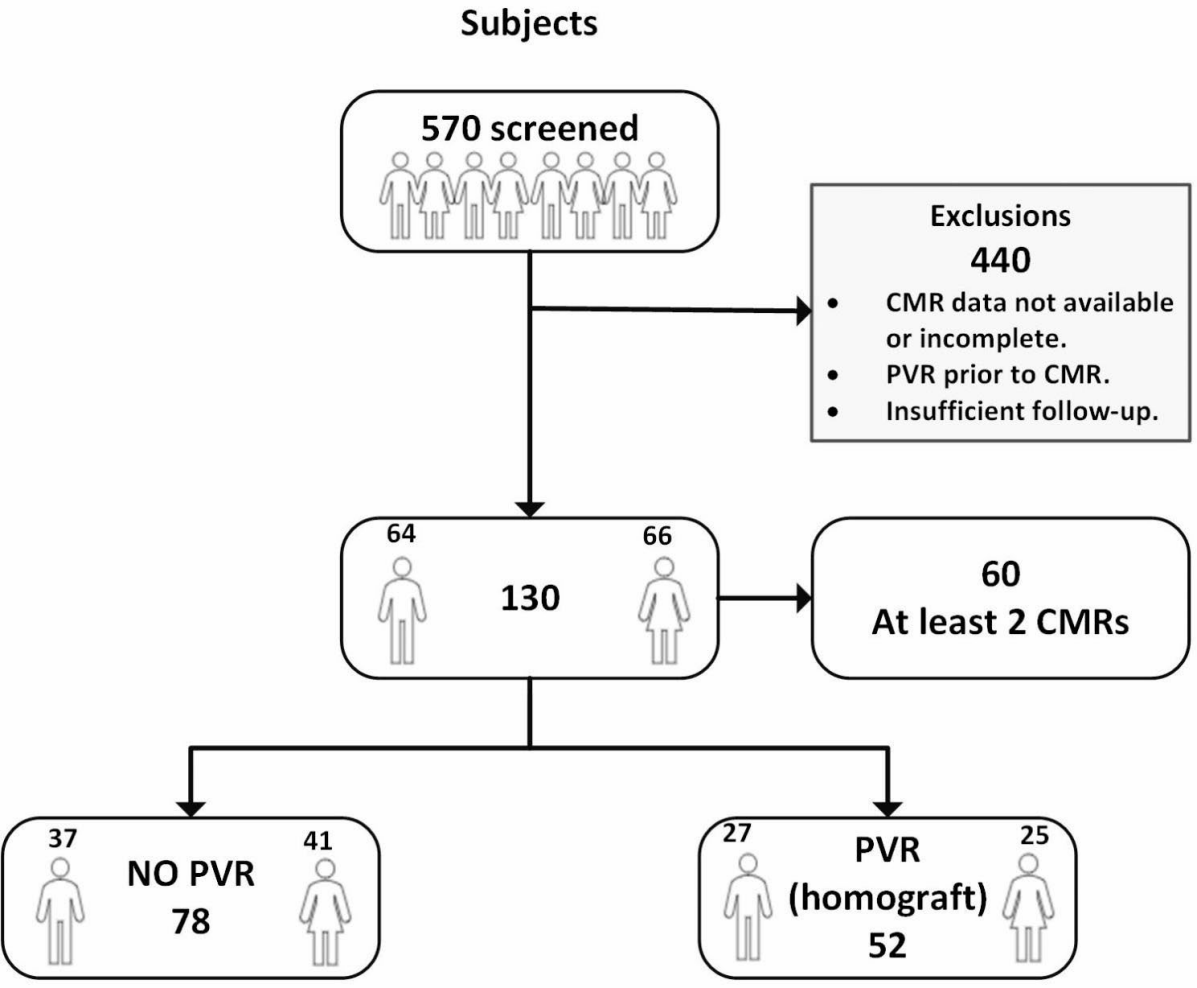
Study enrolment. LEGEND: Of the 570 subjects screened for enrolment, 130 fulfilled entry criteria. Among these, 52 underwent surgical PVR (homograft) within the observation period. 440 were excluded because of not available or incomplete CMR data, PVR prior to CMR or insufficient follow-up. In the 60 subjects who performed at least two CMRs during the observation period before eventual PVR, evaluation of ventricular volume increase over time was feasible. CMR = cardiac magnetic resonance; PVR = pulmonary valve replacement

Of the 570 subjects screened for enrolment, 130 (median age 22 years, interquartile range 18–37 years, 66 females) fulfilled entry criteria (Fig. 5). In the 60 subjects who performed at least two CMRs during the observation period before eventual PVR, evaluation of ventricular volume increase over time was feasible. Demographic, anatomic and surgical characteristics stratified by composite outcome are listed in Table 1. Among the 130 cohort patients, 88 experienced the composite outcome. Median follow-up from first CMR was 110.4 months (70.5 to 149.4 months).

**Table 1:** Demographic, anatomic and surgical characteristics of patients stratified by composite outcome

Variable	No outcome (N = 42)		Outcome (N = 88)		All patients (N = 130)		p
**Gender, male**	16	(38.1%)	48	(54.5%)	64	(49.2%)	0.079
**Body mass index (kg/m2)**	21.8	(20.0 -24.4)	23.1	(21.1–25.2)	22.6	(20.7–25.0)	0.127
**Body surface area (m2)**	1.7	(1.5–1.9)	1.7	(1.6–1.8)	1.7	(1.6–1.8)	0.811
**Weight (kg)**	60.0	(53.0–75.0)	64.0	(55.5–71.5)	64.0	(55.0–73.0)	0.424
**Height (m)**	1.7	(1.6–1.7)	1.7	(1.6–1.7)	1.7	(1.6–1.7)	0.657
**Age at first CMR (years)**	18.5	(17.0 -20.9)	27.9	(19.2–43.0)	22.1	(18.0 -36.7)	< 0.001
**Age at TOF repair (months), N = 127**	12.6	(7.4–27.0)	30.1	(11.4–88.9)	23.1	(9.5–67.7)	0.001
**Time from repair to CMR (years), N = 127**	17.1	(16.2–19.1)	23.5	(17.4–33.2)	19.1	(16.8–28.5)	< 0.001
**Follow-up time after first CMR (months)**	85.2	(46.0 -125.6)	125.8	(81.3 -158.5)	110.4	(70.5 -149.4)	0.002
**Time without arrhythmias (months), N = 116**	65.5	(33.9–87.5)	64.0	(30.4 -118.6)	64.0	(32.3 -101.8)	0.816
**Type of TOF repair**							0.461
Transannular patch	26	(61.9%)	54	(61.4%)	80	(61.5%)	
Infundibular patch	14	(33.3%)	21	(23.9%)	35	(26.9%)	
Double revision of repair	1	(2.4%)	5	(5.7%)	6	(4.6%)	
Other	0	(0.0%)	2	(2.3%)	2	(1.5%)	
Unknown	1	(2.4%)	6	(6.8%)	7	(5.4%)	
**Pulmonary valve reintervention**							0.001
No reintervention	31	(73.8%)	39	(44.3%)	70	(53.8%)	
Homograft	7	(16.7%)	45	(51.1%)	52	(40.0%)	
Valvuloplasty	1	(2.4%)	0	(0.0%)	1	(0.8%)	
PPVI	3	(7.1%)	4	(4.5%)	7	(5.4%)	
**PA procedures pre-CMR**							0.108
None	34	(81.0%)	79	(89.8%)	113	(86.9%)	
Monolateral PA stent(s)	6	(14.3%)	3	(3.4%)	9	(6.9%)	
Bilateral PA stents	1	(2.4%)	1	(1.1%)	2	(1.5%)	
PTA	1	(2.4%)	5	(5.7%)	6	(4.6%)	
**PA procedures post-CMR pre-homograft**							0.552
None	41	(97.6%)	81	(92.0%)	122	(93.8%)	
Monolateral PA stent(s)	1	(2.4%)	3	(3.4%)	4	(3.1%)	
Bilateral PA stents	0	(0.0%)	2	(2.3%)	2	(1.5%)	
PTA	0	(0.0%)	2	(2.3%)	2	(1.5%)	
**PA procedures post-homograft**							0.616
None	42	(100.0%)	86	(97.7%)	128	(98.5%)	
Homograft PTA	0	(0.0%)	1	(1.1%)	1	(0.8%)	
Homograft stent and PTA	0	(0.0%)	1	(1.1%)	1	(0.8%)	

CMR = cardiac magnetic resonance; TOF = Tetralogy of Fallot; PA = pulmonary artery (arteries); PPVI = percutaneous pulmonary valve implantation; PTA = percutaneous transluminal angioplasty

### Outcomes

Multiple CMR and clinical variables correlated to the composite outcome and to the single outcomes separately on both univariate and multivariate analyses (Tables 2, 3 and 4 and Supplementary Tables 1–3 of Additional file 1). RV volumes were adverse outcome predictors at univariate analysis both as continuous and categorical variables (RVEDVi ≥ 160ml/m^2^, RVESVi ≥ 85ml/m^2^ and RVESVi ≥ 65ml/m^2^). RVESVi ≥ 65ml/m^2^ was included in the best predictive model for QRS ≥ 160ms by multivariate analysis. Abnormal IVS motion (diastolic flattening, systo-diastolic flattening, or diastolic inversion), its duration and extent were associated with the composite outcome and abnormal IVS motion was also associated with HF hospitalization and arrhythmias as single outcomes. Angled MPA bifurcation was related to the composite outcome and to arrhythmias, pharmacological therapy, HF hospitalization and VO_2_max reduction ≥ 5% as single outcomes. RPA and LPA dilation are both associated with the composite outcome. RPA dilation is also related to the need for pharmacological therapy. The increasing number of stenotic, hypoplastic or dilated pulmonary arteries (RPA and LPA: none of them, only one, both) is associated to the composite outcome and the need for pharmacological therapy. In subjects with RVOT aneurysm, the coronal diameter of the aneurysm was associated with NYHA class increase, HF hospitalization, need for pharmacological therapy, RVEDVi increase > 20ml/m^2^ and RVESVi increase > 20ml/m^2^. The sagittal diameter of RVOT aneurysm was related to QRS ≥ 160ms and HF hospitalization. RVcEF was associated with the composite outcome and QRS ≥ 160ms while IVS LGE was associated with arrhythmias and pharmacological therapy. RA dimensions (area, latero-lateral and apicobasal diameter) were associated with the composite outcome and most of the single adverse outcomes separately on both univariate and multivariate analyses. Especially RA dimensions were adverse outcomes’ predictors for arrhythmias, QRS ≥ 160ms, HF hospitalization, NYHA class increase, need for pharmacological therapy, and RA area was included in the best predictive model for QRS ≥ 160ms by multivariate analysis. Age at first CMR, age at TOF repair, time from repair to CMR and type of TOF repair were the clinical parameters most associated with adverse outcomes. Multivariate analysis allowed to develop predictive models for QRS ≥ 160ms and the need of pharmacological therapy (Table 5).

**Table 2:** CMR and clinical variables correlated to the composite outcome

Variable	No outcome (N = 42)		Outcome (N = 88)		All patients (N = 130)		p
**LV mass index (g/m2)**	42.0	(37.0–49.0)	46.0	(40.0–54.0)	44.0	(39.0–52.0)	0.032
**LV mass/volume (g/ml)**	0.5	(0.5–0.6)	0.6	(0.5–0.7)	0.6	(0.5–0.7)	0.007
**RV EDVi (ml/m2)**	112.5	(95.0 -139.0)	132.0	(111.5 -153.5)	128.5	(105.0 -148.0)	0.006
**RV ESVi (ml/m2)**	55.0	(44.0–65.0)	66.0	(53.0 -80.5)	64.5	(47.0–76.0)	0.002
**RV EDV (ml)**	202.6	(161.4 -224.4)	219.2	(189.0 -272.2)	214.2	(178.7 -253.4)	0.011
**RV ESV (ml)**	97.4	(73.5 -113.5)	113.5	(87.3 -139.4)	108.6	(81.6 -129.6)	0.006
**RV cEF (%), N = 127**	31.5	(27.3–46.0)	28.6	(22.6–36.0)	29.8	(23.9–38.0)	0.016
**RA transverse diameter (cm)**	4.5	(3.8–5.1)	5.2	(4.5–5.8)	4.9	(4.3–5.5)	< 0.001
**RA longitudinal diameter (cm)**	4.9	(4.1–5.2)	5.2	(4.5 -6.0)	5.0	(4.4–5.6)	0.005
**RA area (cm2)**	17.5	(15.0–22.0)	22.5	(18.0 -28.5)	20.0	(16.0–25.0)	< 0.001
**Maximum IVS flattening angle (°), N = 73**	145.0	(134.0 -149.0)	150.0	(140.0 -185.0)	147.0	(138.0 -162.0)	0.016
**Pulmonary regurgitation (ml/beat), N = 124**	32.0	(13.0–50.0)	46.0	(25.0–61.0)	41.0	(22.5–55.0)	0.016
**Pulmonary regurgitation (%), N = 127**	39.0	(14.0–48.0)	43.0	(31.0–53.0)	41.0	(27.0–50.0)	0.045
**RVEDVi increase (ml/m2), N = 60**	1.0	(-5.0 -15.0)	17.0	(0.0–25.0)	9.5	(-3.5 -23.0)	0.009
**RVESVi increase (ml/m2), N = 60**	-1.0	(-2.0 -7.0)	12.0	(0.0–24.0)	9.0	(-2.0 -17.5)	0.010
**Age at first CMR (years)**	18.5	(17.0 -20.9)	27.9	(19.2–43.0)	22.1	(18.0 -36.7)	< 0.001
**Age at TOF repair (months), N = 127**	12.6	(7.4–27.0)	30.1	(11.4–88.9)	23.1	(9.5–67.7)	0.001
**Time from repair to CMR (years), N = 127**	17.1	(16.2–19.1)	23.5	(17.4–33.2)	19.1	(16.8–28.5)	< 0.001
**Follow-up time after first CMR (months)**	85.2	(46.0 -125.6)	125.8	(81.3 -158.5)	110.4	(70.5 -149.4)	0.002
**Pulmonary valve reintervention**							0.001
No reintervention	31	(73.8%)	39	(44.3%)	70	(53.8%)	
Homograft	7	(16.7%)	45	(51.1%)	52	(40.0%)	
Valvuloplasty	1	(2.4%)	0	(0.0%)	1	(0.8%)	
PPVI	3	(7.1%)	4	(4.5%)	7	(5.4%)	
**RV EDVi ≥ 160 ml/m2**							0.040
No	39	(92.9%)	69	(78.4%)	108	(83.1%)	
Yes	3	(7.1%)	19	(21.6%)	22	(16.9%)	
**RV ESVi ≥ 65 ml/m2**							0.009
No	28	(66.7%)	37	(42.0%)	65	(50.0%)	
Yes	14	(33.3%)	51	(58.0%)	65	(50.0%)	
**RV ESVi ≥ 85 ml/m2**							0.005
No	41	(97.6%)	69	(78.4%)	110	(84.6%)	
Yes	1	(2.4%)	19	(21.6%)	20	(15.4%)	
**Type of abnormal IVS motion**							0.009
Normal IVS motion	23	(54.8%)	34	(38.6%)	57	(43.8%)	
Diastolic IVS flattening	18	(42.9%)	30	(34.1%)	48	(36.9%)	
Systo-diastolic IVS flattening	0	(0.0%)	4	(4.5%)	4	(3.1%)	
Diastolic IVS inversion	1	(2.4%)	20	(22.7%)	21	(16.2%)	
**Abnormal IVS motion duration**							0.033
None	23	(54.8%)	34	(38.6%)	57	(43.8%)	
Protodiastolic	9	(21.4%)	12	(13.6%)	21	(16.2%)	
Holodiastolic	10	(23.8%)	42	(47.7%)	52	(40.0%)	
**Abnormal IVS motion extent**							0.033
Normal IVS motion	23	(54.8%)	34	(38.6%)	57	(43.8%)	
Basal	2	(4.8%)	3	(3.4%)	5	(3.8%)	
Basal-middle	4	(9.5%)	2	(2.3%)	6	(4.6%)	
Basal-middle-apical	13	(31.0%)	49	(55.7%)	62	(47.7%)	
**RPA, N = 129**							0.010
Normal anatomy	33	(80.5%)	49	(55.7%)	82	(63.6%)	
Stenosis	4	(9.8%)	11	(12.5%)	15	(11.6%)	
Hypoplasia	2	(4.9%)	2	(2.3%)	4	(3.1%)	
Dilation	2	(4.9%)	26	(29.5%)	28	(21.7%)	
**LPA, N = 129**							0.031
Normal anatomy	20	(48.8%)	28	(31.8%)	48	(37.2%)	
Stenosis	12	(29.3%)	21	(23.9%)	33	(25.6%)	
Hypoplasia	4	(9.8%)	6	(6.8%)	10	(7.8%)	
Dilation	5	(12.2%)	33	(37.5%)	38	(29.5%)	
**MPA bifurcation geometry**							0.039
Normal	38	(90.5%)	66	(75.0%)	104	(80.0%)	
Angled	4	(9.5%)	22	(25.0%)	26	(20.0%)	
**Number of stenotic/hypoplastic/dilated PA, N = 129**							0.005
0	17	(41.5%)	25	(28.4%)	42	(32.6%)	
1	19	(46.3%)	27	(30.7%)	46	(35.7%)	
2	5	(12.2%)	36	(40.9%)	41	(31.8%)	

LV = left ventricle; RVEDVi = right ventricle end-diastolic volume indexed to BSA; RVESVi = right ventricle end-systolic volume indexed to BSA; RVEDV = right ventricle end-diastolic volume; RVESV = right ventricle end-systolic volume; RVcEF = right ventricle ejection fraction corrected for pulmonary regurgitation; RA = right atrium; IVS = interventricular septum; CMR = cardiac magnetic resonance; TOF = Tetralogy of Fallot; PPVI = percutaneous pulmonary valve implantation; RPA = right pulmonary artery; LPA = left pulmonary artery; MPA = main pulmonary artery; PA = pulmonary artery (arteries)

**Table 3:** CMR and clinical variables correlated to the need of pharmacological therapy

Variable	No outcome (N = 93)		Outcome (N = 37)		All patients (N = 130)		p
**VO2max (ml/kg/min), N = 71**	29.0	(27.1–34.1)	23.9	(17.3–31.1)	28.0	(23.9–32.5)	0.002
**VO2max pre-homograft (ml/kg/min), N = 38**	29.5	(24.0 -32.4)	20.2	(15.4–22.2)	27.0	(20.8–31.0)	0.006
**Body mass index (kg/m2)**	22.1	(20.3–24.2)	24.2	(22.6–26.2)	22.6	(20.7–25.0)	0.005
**Weight (kg)**	60.0	(55.0–70.0)	68.0	(60.0–78.0)	64.0	(55.0–73.0)	0.041
**LV mass/volume (g/ml)**	0.6	(0.5–0.6)	0.6	(0.5–0.7)	0.6	(0.5–0.7)	0.025
**RA transverse diameter (cm)**	4.7	(4.2–5.2)	5.5	(4.7–6.7)	4.9	(4.3–5.5)	< 0.001
**RA longitudinal diameter (cm)**	4.9	(4.2–5.3)	5.7	(5.0 -6.9)	5.0	(4.4–5.6)	< 0.001
**RA area (cm2)**	19.0	(16.0–23.0)	27.0	(22.0–36.0)	20.0	(16.0–25.0)	< 0.001
**IVS LGE (cm)**	0.0	(0.0–0.0)	0.0	(0.0 -2.2)	0.0	(0.0 -1.5)	0.009
**RVOT aneurysm coronal diameter (mm), N = 32**	34.0	(32.0–36.0)	43.0	(38.0–54.0)	35.5	(33.5–42.5)	0.003
**Age at first CMR (years)**	19.9	(17.6–25.1)	43.9	(34.9–50.6)	22.1	(18.0 -36.7)	< 0.001
**Age at TOF repair (months), N = 127**	14.7	(7.6–34.5)	93.6	(36.5 -170.2)	23.1	(9.5–67.7)	< 0.001
**Time from repair to CMR (years), N = 127**	17.9	(16.7–21.5)	32.6	(21.0 -41.2)	19.1	(16.8–28.5)	< 0.001
**Type of TOF repair**							0.048
Transannular patch	62	(66.7%)	18	(48.6%)	80	(61.5%)	
Infundibular patch	25	(26.9%)	10	(27.0%)	35	(26.9%)	
Double revision of repair	3	(3.2%)	3	(8.1%)	6	(4.6%)	
Other	1	(1.1%)	1	(2.7%)	2	(1.5%)	
Unknown	2	(2.2%)	5	(13.5%)	7	(5.4%)	
**IVS LGE**							0.018
No	72	(77.4%)	21	(56.8%)	93	(71.5%)	
Yes	21	(22.6%)	16	(43.2%)	37	(28.5%)	
**RPA, N = 129**							0.044
Normal anatomy	64	(69.6%)	18	(48.6%)	82	(63.6%)	
Stenosis	11	(12.0%)	4	(10.8%)	15	(11.6%)	
Hypoplasia	3	(3.3%)	1	(2.7%)	4	(3.1%)	
Dilation	14	(15.2%)	14	(37.8%)	28	(21.7%)	
**MPA bifurcation geometry**							0.007
Normal	80	(86.0%)	24	(64.9%)	104	(80.0%)	
Angled	13	(14.0%)	13	(35.1%)	26	(20.0%)	
**Sinus rhythm**							0.001
No	0	(0.0%)	4	(10.8%)	4	(3.1%)	
Yes	93	(100.0%)	33	(89.2%)	126	(96.9%)	
**NYHA class, N = 129**							0.012
1	84	(90.3%)	25	(69.4%)	109	(84.5%)	
2	8	(8.6%)	7	(19.4%)	15	(11.6%)	
3	1	(1.1%)	3	(8.3%)	4	(3.1%)	
4	0	(0.0%)	1	(2.8%)	1	(0.8%)	
**Number of stenotic/hypoplastic/dilated PA, N = 129**							0.021
0	35	(38.0%)	7	(18.9%)	42	(32.6%)	
1	34	(37.0%)	12	(32.4%)	46	(35.7%)	
2	23	(25.0%)	18	(48.6%)	41	(31.8%)	

LV = left ventricle; RA = right atrium; IVS = interventricular septum; LGE = late Gadolinium enhancement; RVOT = right ventricular outflow tract; CMR = cardiac magnetic resonance; TOF = Tetralogy of Fallot; RPA = right pulmonary artery; MPA = main pulmonary artery; NYHA = New York Heart Association; PA = pulmonary artery (arteries)

**Table 4:** CMR and clinical variables correlated to QRS ≥ 160ms

Variable	No outcome (N = 85)		Outcome (N = 45)		All patients (N = 130)		p
**Body surface area (m2)**	1.7	(1.5–1.8)	1.8	(1.6–1.9)	1.7	(1.6–1.8)	0.023
**Weight (kg)**	60.0	(52.0–70.0)	68.0	(60.0–77.0)	64.0	(55.0–73.0)	0.017
**RV EDVi (ml/m2)**	120.0	(98.0 -144.0)	134.0	(116.0 -172.0)	128.5	(105.0 -148.0)	0.025
**RV ESVi (ml/m2)**	58.0	(44.0–74.0)	69.0	(57.0–93.0)	64.5	(47.0–76.0)	0.001
**RV EDV (ml)**	200.7	(172.9 -239.1)	234.6	(194.9 -278.1)	214.2	(178.7 -253.4)	0.003
**RV ESV (ml)**	97.4	(76.0 -118.1)	119.0	(97.4 -156.2)	108.6	(81.6 -129.6)	< 0.001
**RV EF (%)**	52.0	(46.0–57.0)	48.0	(45.0–51.0)	50.0	(45.0–56.0)	0.004
**RV cEF (%), N = 127**	30.4	(25.5–39.3)	27.4	(21.4–33.7)	29.8	(23.9–38.0)	0.034
**RA transverse diameter (cm)**	4.6	(4.1–5.3)	5.2	(4.6–5.7)	4.9	(4.3–5.5)	0.003
**RA longitudinal diameter (cm)**	4.9	(4.2–5.5)	5.2	(4.6 -6.0)	5.0	(4.4–5.6)	0.027
**RA area (cm2)**	19.0	(15.0–23.0)	23.0	(20.0–27.0)	20.0	(16.0–25.0)	0.001
**RVOT aneurysm sagittal diameter (mm), N = 32**	28.0	(25.0–37.0)	40.0	(33.0–43.0)	32.0	(25.5–39.0)	0.006
**Age at first CMR (years)**	19.9	(17.3–33.5)	29.1	(20.2–37.1)	22.1	(18.0 -36.7)	0.006
**Age at TOF repair (months), N = 127**	15.5	(9.0 -45.3)	35.8	(11.4–88.9)	23.1	(9.5–67.7)	0.040
**Time from repair to CMR (years), N = 127**	17.8	(15.9–25.9)	23.6	(18.3–31.4)	19.1	(16.8–28.5)	0.005
**Follow-up time after first CMR (months)**	91.5	(61.3 -140.3)	129.1	(86.5 -160.4)	110.4	(70.5 -149.4)	0.011
**Gender**							0.012
Female	50	(58.8%)	16	(35.6%)	66	(50.8%)	
Male	35	(41.2%)	29	(64.4%)	64	(49.2%)	
**RV EDVi ≥ 160 ml/m2**							0.031
No	75	(88.2%)	33	(73.3%)	108	(83.1%)	
Yes	10	(11.8%)	12	(26.7%)	22	(16.9%)	
**RV ESVi ≥ 65 ml/m2**							0.006
No	50	(58.8%)	15	(33.3%)	65	(50.0%)	
Yes	35	(41.2%)	30	(66.7%)	65	(50.0%)	
**RV ESVi ≥ 85 ml/m2**							0.002
No	78	(91.8%)	32	(71.1%)	110	(84.6%)	
Yes	7	(8.2%)	13	(28.9%)	20	(15.4%)	

RVEDVi = right ventricle end-diastolic volume indexed to BSA; RVESVi = right ventricle end-systolic volume indexed to BSA; RVEDV = right ventricle end-diastolic volume; RVESV = right ventricle end-systolic volume; RVEF = right ventricle ejection fraction; RVcEF = right ventricle ejection fraction corrected for pulmonary regurgitation; RA = right atrium; RVOT = right ventricular outflow tract; CMR = cardiac magnetic resonance; TOF = Tetralogy of Fallot

**Table 5:** Best predictive models for QRS ≥ 160ms and pharmacological therapy by multivariate analysis

All patients (N = 130)	OR	95% CI	p	AUC	95% CI AUC	H-L test *p*-value
**Model “QRS ≥ 160ms” (45 outcomes)**				0.74	(0.65 to 0.83)	0.9082
RVESVi ≥ 65 ml/m2	2.85	(1.25 to 6.50)	0.013			
RA area (cm2)	1.06	(1.01 to 1.11)	0.021			
Male	3.12	(1.38 to 7.07)	0.006			
**Model “Pharmacological therapy” (37 outcomes)**				0.89	(0.82 to 0.96)	0.7526
Time from repair to CMR (years)	1.10	(1.04 to 1.16)	0.001			
Age at TOF repair (months)	1.02	(1.01 to 1.03)	< 0.001			
IVS LGE (cm)	1.47	(1.04 to 2.10)	0.031			

RVESVi = right ventricle end-systolic volume indexed to BSA; RA = right atrium; CMR = cardiac magnetic resonance; TOF = Tetralogy of Fallot; IVS = interventricular septum; LGE = late Gadolinium enhancement

## Discussion

Despite extensive research, the optimal timing for PVR in adults with rTOF remains uncertain. Studies have identified preoperative RV volumes that would result in normalized postoperative volumes, typically defined as RVEDVi ≥ 160 ml/m2 and RVESVi ≥ 80 ml/m2 [5, 6], although these values may vary slightly between studies [12–16]. However, it is unclear whether meeting these values translates into clinical benefit. This study confirmed the association of greater RV volumes with adverse outcomes and outlined the predictive role of a lower cut-off value, namely RVESVi ≥ 65ml/m^2^, suggesting that even an earlier PVR timing could be beneficial. RVESVi is a highly prognostic indicator of ventricular function and, compared to RVEDVi, more accurately reflects the effects on the ventricle of an increased afterload [17]. Therefore, RVESVi is an important parameter to consider in the therapeutic management of rTOF. Additionally, to define the optimal PVR timing and therapeutic management of these patients, it would be necessary to consider other parameters, in addition to RV volumes, as suggested by the results of this study. Since major arrhythmias are the main cause of sudden cardiac death in rTOF patients, the association of abnormal IVS motion with the development of major arrhythmias could provide useful insights into risk stratification. Threshold values for IVS flattening angles could also help for a better definition of PVR timing. Abnormal IVS motion predicts HF hospitalization as long as it reflects RV pressure and/or volume overload [18, 19]. Optimal branching angles of pulmonary arteries (PAs) fulfil the principle of minimum work which is important especially in congenital heart diseases, where small improvements in the physiological efficiency may result in large effects on mid- and long-term outcome parameters, such as longer lasting RV function or appropriate growth of the PAs [11]. MPA angled bifurcation impairs PAs hemodynamics causing RV overload and could lead to typical dysfunctions requiring specific therapeutic timings and solutions. RVOT aneurysm diameters are associated with adverse outcomes mainly related to RV volume increase and HF, probably because RVOT dilation adversely affects RV hemodynamics and systolic function. RVOT aneurysm relates to surgical repair through patching, post-surgical scarring and altered electrical impulse conduction. In fact, RVOT aneurysm sagittal diameter is also related to QRS ≥ 160ms. The importance of this association lies in the increased risk of major arrhythmias in subjects with elongated QRS [20]. RVOT diameters threshold values could be useful for a better definition of PVR timing. RVcEF association with adverse outcomes and its greater post-PVR increase compared to RVEF underline its potential role for a better evaluation of RV functional recovery. RVcEF reflects RV contractile and mechanical function, but also its actual pulmonary blood outflow. The association of IVS LGE with arrhythmias and pharmacological therapy is probably due respectively to altered electrical impulse conduction and contractile impairment induced by myocardial fibrosis [20, 21]. The stronger statistical significance of IVS LGE extent, as opposed to its mere presence or absence, highlights the importance of quantitative or semi-quantitative fibrosis assessment, and not just qualitative assessment, as already demonstrated in hypertrophic cardiomyopathy [22]. The recurrence of RA dimensions (area, transverse diameter, longitudinal diameter) as strong outcomes’ predictors suggests the importance of RV diastolic function in assessing the evolution towards ventricular dysfunction. By enabling straightforward and repeatable measurement of these highly informative parameters, CMR represents the standard of excellence for evaluating the right heart sections. RV hypertrophy (RV mass index) is also related to the risk of HF hospitalization as it may be due to prevalent pressure overload, persistence of pulmonary artery stenosis and pulmonary hypertension [2]. The analysis highlights the relevance of LV morpho-functional parameters alongside those pertaining the RV. This is probably due to ventriculo-ventricular interaction as previously described in pulmonary hypertension [23, 24]. Age at TOF repair, type of TOF repair (transannular patch), age at first CMR and time from repair to CMR are the clinical parameters most associated with adverse outcomes. Older age at TOF repair is a well-known predictor of adverse outcomes as it causes prolonged ventricular volume overload and hypoxia, leading to a more severe ventricular dysfunction [25]. Patients who develop adverse outcomes usually have their first CMR later and after a longer interval after TOF repair. Therefore, it might be appropriate to perform TOF repair as early as possible (in the first months/years of life) and to start CMR follow-up in 20-year-olds, intensifying the surveillance 20 years after surgical repair. While the timing of PVR is an important consideration for the management of patients with rTOF, it is also crucial to define the timing of surgical repair and follow-up MRI scans to ensure optimal therapeutic management and the prevention of complications. Technological advancements in MRI and better standardization of mapping techniques may also help to define the effects of overload on rTOF patients and the timing of interventions thanks to a more comprehensive assessment of cardiac function and a more accurate assessment of changes over time.

### Study limitations

Study limitations were the retrospective and monocentric design. Moreover, the cohort is restricted to subjects who have undergone CMR; this limitation is partially mitigated by the routine use of CMR in this patient group at our center. The analyses were also limited by the heterogeneity of TOF population: in future studies it could be useful to stratify this population according to the different morphological (e.g., PAs anatomy) and clinical (e.g., time from surgical repair) characteristics to highlight the differences between subgroups and to understand the optimal management of each. Multicenter prospective studies could be helpful in better defining the role of each of these parameters in the management of rTOF patients.

## Conclusions

In conclusion, the study provides a comprehensive analysis of a large cohort of rTOF subjects. RV volumes significance in determining PVR timing is confirmed and insights on novel clinical and morpho-functional parameters associated with adverse outcomes are provided. These parameters, such as abnormal IVS motion, PAs morphology, RVOT aneurysm, RVcEF, and IVS LGE, are related to RV mechanical stress, dysfunction, and arrhythmogenicity. Importantly, they are easy to calculate and could be readily incorporated into clinical practice. These findings deserve further validation in larger populations.

### Electronic supplementary material

Below is the link to the electronic supplementary material.

Additional file 1. Materials and methods; Supplementary tables. Description of data: In “Additional file 1” supplementary information is provided for the Data collection section of Methods (Patient demographics and clinical data, CMR imaging, Electrocardiographic data, Exercise Testing). “Additional file 1” also contains Supplementary tables 1-3.

## Data Availability

The datasets used and/or analyzed during the current study are available from the corresponding author on reasonable request.

## Abbreviations

ACHD: Adult congenital heart disease
AUC: Area under the ROC curve
BMI: Body mass index
BSA: Body surface area
bSSFP: Balanced steady-state free precession
CE-AVEC: Comitato Etico-Area Vasta Emilia Centro
ceMRA: Contrast enhanced magnetic resonance angiography
CHD: Congenital heart disease
CMR: Cardiac magnetic resonance
EDV: End-diastolic volume
EF: Ejection fraction
ESV: Eend-systolic volume
HF: Heart failure
IR-FGRE: Inversion recovery fast gradient echo
IVS: Interventricular septum
LGE: Late Gadolinium enhancement
LPA: Left pulmonary artery
LV: Left ventricle
LVEF: Left ventricle ejection fraction
LVEDV: Left ventricle end-diastolic volume
LVEDVi: Left ventricle end-diastolic volume indexed to BSA
LVESV: Left ventricle end-systolic volume
LVESVi: Left ventricle end-systolic volume indexed to BSA
MIP: Maximum intensity projection
MPA: Main pulmonary artery
netPFV: Net Pulmonary Forward Volume
NYHA: New York Heart Association
PA: Pulmonary artery (arteries)
PC: Phase contrast
PPVI: Percutaneous pulmonary valve implantation
PR: Pulmonary regurgitation
PRF: Pulmonary regurgitant fraction
PTA: Percutaneous transluminal angioplasty
PVR: Pulmonary valve replacement
RA: Right atrium
ROC: Receiver Operator Characteristic
H-L: Hosmer-Lemeshow
rTOF: Repaired Tetralogy of Fallot
RPA: Right pulmonary artery
RV: Right ventricle
RVcEF: Right ventricle ejection fraction corrected for pulmonary regurgitation
RVEF: Right ventricle ejection fraction
RVEDV: Right ventricle end-diastolic volume
RVEDVi: Right ventricle end-diastolic volume indexed to BSA
RVESV: Right ventricle end-systolic volume
RVESVi: Right ventricle end-systolic volume indexed to BSA
RVOT: Right ventricle outflow tract
SV: Stroke volume
SVT: Supraventricular tachycardia
TAPSE: Tricuspid annular plane systolic excursion
TOF: Tetralogy of Fallot
VT: Ventricular tachycardia
