# Supplementary material for: Predictive value of Cardiac Magnetic Resonance: new and old parameters in the natural history of repaired Tetralogy of Fallot
Source: BMC Cardiovasc Disord. 2024 Jan 3;24:15. doi: 10.1186/s12872-023-03671-4 (PMC10765701; doi:10.1186/s12872-023-03671-4)
Supplement: Supplementary file 1 — Additional file 1. Materials and methods; Supplementary tables. Description of data: In “Additional file 1” supplementary information is provided for the Data collection section of Methods (Patient demographics and clinical data, CMR imaging, Electrocardiographic data, Exercise Testing). “Additional file 1” also contains Supplementary tables 1-3. [file 12872_2023_3671_MOESM1_ESM.docx]

**MATERIALS AND METHODS**

**Data collection**

**Patient demographics and clinical data**

Data collected included date of birth, gender, weight (kg), height (m), Body Surface Area (m^2^, BSA), Body Mass Index (kg/m^2^, BMI), age at TOF repair (months), type of TOF repair (classified as transannular patch, infundibular patch, double revision of repair, other/unknown), age at first CMR (years), time from TOF repair to CMR (years), follow-up time after first CMR (months), age at first pulmonary valve reintervention, type of first pulmonary valve reintervention (classified as no reintervention, homograft, valvuloplasty and percutaneous pulmonary valve implantation (PPVI)), date and type of pulmonary arteries (PA) procedures pre-CMR (classified as none, monolateral PA stent(s), bilateral PA stents, percutaneous transluminal angioplasty (PTA), homograft PTA, homograft stent and PTA), date and type of PA procedures post-CMR pre-homograft, date and type of PA procedures post-homograft.

Clinical history and dates of any arrhythmias, time from the first CMR without any arrhythmias (months), NYHA class, episodes of heart failure that needed hospitalization, need for pharmacological therapy assumption and sudden cardiac death were recorded.

**CMR imaging**

Ventricular volumes, mass and function: End-systolic and end-diastolic phases of the cardiac cycle were determined visually.

LV papillary muscles and RV major trabeculations were included in the blood pool and not considered part of the myocardial mass measured in end-diastole.

RV and LV endocardial and epicardial contours were traced in end-diastole; endocardial contours alone were traced in systole.

The RV and LV outflow tracts (to the level of the semilunar valves) were included in respective ventricular volumes.

LV and RV end-diastolic volume (EDV), end-systolic volume (ESV), stroke volume (SV), and mass were measured and adjusted to BSA (LVEDVi and LVESVi; RVEDVi and RVESVi).

RVEDVi and RVESVi variations between the first and last MRIs performed within the observation period, before eventual PVR, were also calculated.

LV and RV ejection fractions (EF) were calculated as 100*(EDV - ESV)/EDV.

**Electrocardiographic data**

The resting ECG performed closest to the first CMR was reviewed, and any cardiac rhythm abnormalities together with the resting heart rate (bpm) were recorded. All arrhythmic episodes reported in the patients' medical records occurred during the follow-up period were noted with date. The most recent resting ECG performed during the observation period (before pulmonary valve reintervention, if performed) was reviewed for evidence of QRS duration greater than or equal to 160ms.

**Exercise Testing**

The type of exercise test was treadmill or cycle ergometer. The peak oxygen consumption (VO_2_max (ml/kg/min)) and its percent predicted value for age and sex at the time of the first and last CMR performed within the observation period, before eventual PVR, were recorded when available.

**SUPPLEMENTARY TABLES**

**Supplementary table 1**

CMR and clinical variables correlated to arrhythmias.

| **Variable** | **No outcome (N=116)** | | | **Outcome (N=14)** | | **All patients (N=130)** | | **p** |
| --- | --- | --- | --- | --- | --- | --- | --- | --- |
| **Age at first CMR (years)** | | 20.6 | (17.8 -32.5) | 41.9 | (34.9 -47.8) | 22.1 | (18.0 -36.7) | <0.001 |
| **Age at TOF repair (months), N=127** | | 20.2 | (9.0 -48.1) | 74.8 | (46.5 -104.1) | 23.1 | (9.5 -67.7) | 0.006 |
| **Time from repair to CMR (years), N=127** | | 18.3 | (16.5 -25.9) | 33.2 | (31.3 -38.1) | 19.1 | (16.8 -28.5) | <0.001 |
| **Follow-up time after first CMR (months)** | | 98.7 | (62.5 -145.3) | 133.8 | (114.9 -164.9) | 110.4 | (70.5 -149.4) | 0.018 |
| **VO2max pre-homograft (ml/kg/min), N=38** | | 28.5 | (22.2 -31.7) | 17.2 | (13.0 -22.9) | 27.0 | (20.8 -31.0) | 0.020 |
| **RA transverse diameter (cm)** | | 4.8 | (4.2 -5.5) | 5.5 | (5.0 -6.5) | 4.9 | (4.3 -5.5) | 0.004 |
| **RA longitudinal diameter (cm)** | | 5.0 | (4.2 -5.5) | 5.5 | (5.0 -6.9) | 5.0 | (4.4 -5.6) | 0.023 |
| **RA area (cm2)** | | 20.0 | (16.0 -24.0) | 26.0 | (23.0 -35.0) | 20.0 | (16.0 -25.0) | 0.001 |
| **IVS LGE (cm)** | | 0.0 | (0.0 -1.2) | 0.8 | (0.0 -2.2) | 0.0 | (0.0 -1.5) | 0.050 |
| **End-diastolic IVS flattening angle (°), N=73** | | 139.0 | (133.0 -146.0) | 150.0 | (139.0 -164.0) | 140.0 | (133.0 -146.0) | 0.011 |
| **Maximum IVS flattening angle (°), N=73** | | 146.0 | (137.0 -158.0) | 185.0 | (150.0 -202.0) | 147.0 | (138.0 -162.0) | 0.023 |
| **RVESVi increase (ml/m2), N=60** | | 5.5 | (-2.0 -12.5) | 21.0 | (12.0 -24.0) | 9.0 | (-2.0 -17.5) | 0.029 |
| **Type of TOF repair** | |  |  |  |  |  |  | <0.001 |
| Transannular patch | | 76 | (65.5%) | 4 | (28.6%) | 80 | (61.5%) |  |
| Infundibular patch | | 31 | (26.7%) | 4 | (28.6%) | 35 | (26.9%) |  |
| Double revision of repair | | 3 | (2.6%) | 3 | (21.4%) | 6 | (4.6%) |  |
| Other | | 2 | (1.7%) | 0 | (0.0%) | 2 | (1.5%) |  |
| Unknown | | 4 | (3.4%) | 3 | (21.4%) | 7 | (5.4%) |  |
| **PA procedures post-homograft** | |  |  |  |  |  |  | 0.015 |
| None | | 115 | (99.1%) | 13 | (92.9%) | 128 | (98.5%) |  |
| Homograft PTA | | 0 | (0.0%) | 1 | (7.1%) | 1 | (0.8%) |  |
| Homograft stent and PTA | | 1 | (0.9%) | 0 | (0.0%) | 1 | (0.8%) |  |
| **Type of abnormal IVS motion** | |  |  |  |  |  |  | 0.037 |
| Normal IVS motion | | 50 | (43.1%) | 7 | (50.0%) | 57 | (43.8%) |  |
| Diastolic IVS flattening | | 47 | (40.5%) | 1 | (7.1%) | 48 | (36.9%) |  |
| Systo-diastolic IVS flattening | | 3 | (2.6%) | 1 | (7.1%) | 4 | (3.1%) |  |
| Diastolic IVS inversion | | 16 | (13.8%) | 5 | (35.7%) | 21 | (16.2%) |  |
| **MPA bifurcation geometry** | |  |  |  |  |  |  | <0.001 |
| Normal | | 100 | (86.2%) | 4 | (28.6%) | 104 | (80.0%) |  |
| Angled | | 16 | (13.8%) | 10 | (71.4%) | 26 | (20.0%) |  |

CMR= cardiac magnetic resonance; TOF= Tetralogy of Fallot; RA= right atrium; IVS= interventricular septum; LGE= late Gadolinium enhancement; RVESVi= right ventricle end-systolic volume indexed to BSA; PA= pulmonary artery (arteries); PTA= percutaneous transluminal angioplasty; MPA= main pulmonary artery.

**Supplementary table 2**

CMR and clinical variables correlated to heart failure hospitalization.

| **Variable** | **No outcome (N=118)** | | | **Outcome (N=12)** | | **All patients (N=130)** | | **p** |
| --- | --- | --- | --- | --- | --- | --- | --- | --- |
| **LV mass/volume (g/ml)** | | 0.6 | (0.5 -0.7) | 0.6 | (0.6 -0.8) | 0.6 | (0.5 -0.7) | 0.039 |
| **RV EDV (ml)** | | 214.0 | (176.7 -242.5) | 276.7 | (199.0 -312.7) | 214.2 | (178.7 -253.4) | 0.043 |
| **RV ESV (ml)** | | 102.5 | (80.5 -124.6) | 141.8 | (97.6 -186.3) | 108.6 | (81.6 -129.6) | 0.048 |
| **RA transverse diameter (cm)** | | 4.8 | (4.2 -5.4) | 5.8 | (5.3 -6.7) | 4.9 | (4.3 -5.5) | 0.001 |
| **RA longitudinal diameter (cm)** | | 5.0 | (4.2 -5.5) | 6.1 | (5.0 -7.1) | 5.0 | (4.4 -5.6) | 0.010 |
| **RA area (cm2)** | | 20.0 | (16.0 -24.0) | 27.0 | (23.0 -38.5) | 20.0 | (16.0 -25.0) | 0.001 |
| **Maximum IVS flattening angle (°), N=73** | | 146.0 | (137.0 -156.0) | 192.0 | (150.0 -210.0) | 147.0 | (138.0 -162.0) | 0.003 |
| **RVOT aneurysm coronal diameter (mm), N=32** | | 35.0 | (33.0 -41.0) | 53.0 | (51.0 -55.0) | 35.5 | (33.5 -42.5) | 0.040 |
| **RVOT aneurysm sagittal diameter (mm), N=32** | | 31.0 | (25.0 -39.0) | 40.0 | (38.0 -45.0) | 32.0 | (25.5 -39.0) | 0.021 |
| **Age at first CMR (years)** | | 21.1 | (17.9 -35.1) | 45.7 | (23.7 -51.6) | 22.1 | (18.0 -36.7) | 0.015 |
| **Age at TOF repair (months), N=127** | | 20.5 | (9.0 -52.1) | 98.3 | (33.5 -173.9) | 23.1 | (9.5 -67.7) | 0.006 |
| **Time from repair to CMR (years), N=127** | | 18.6 | (16.7 -27.2) | 34.8 | (21.6 -41.5) | 19.1 | (16.8 -28.5) | 0.011 |
| **PA procedures post-homograft** | |  |  |  |  |  |  | 0.007 |
| None | | 117 | (99.2%) | 11 | (91.7%) | 128 | (98.5%) |  |
| Homograft PTA | | 0 | (0.0%) | 1 | (8.3%) | 1 | (0.8%) |  |
| Homograft stent and PTA | | 1 | (0.8%) | 0 | (0.0%) | 1 | (0.8%) |  |
| **LV EF < 50%** | |  |  |  |  |  |  | 0.048 |
| No | | 109 | (92.4%) | 9 | (75.0%) | 118 | (90.8%) |  |
| Yes | | 9 | (7.6%) | 3 | (25.0%) | 12 | (9.2%) |  |
| **RV EDVi >= 160 ml/m2** | |  |  |  |  |  |  | 0.016 |
| No | | 101 | (85.6%) | 7 | (58.3%) | 108 | (83.1%) |  |
| Yes | | 17 | (14.4%) | 5 | (41.7%) | 22 | (16.9%) |  |
| **RV ESVi >= 85 ml/m2** | |  |  |  |  |  |  | 0.008 |
| No | | 103 | (87.3%) | 7 | (58.3%) | 110 | (84.6%) |  |
| Yes | | 15 | (12.7%) | 5 | (41.7%) | 20 | (15.4%) |  |
| **Type of abnormal IVS motion** | |  |  |  |  |  |  | 0.005 |
| Normal IVS motion | | 52 | (44.1%) | 5 | (41.7%) | 57 | (43.8%) |  |
| Diastolic IVS flattening | | 47 | (39.8%) | 1 | (8.3%) | 48 | (36.9%) |  |
| Systo-diastolic IVS flattening | | 4 | (3.4%) | 0 | (0.0%) | 4 | (3.1%) |  |
| Diastolic IVS inversion | | 15 | (12.7%) | 6 | (50.0%) | 21 | (16.2%) |  |
| **MPA bifurcation geometry** | |  |  |  |  |  |  | 0.049 |
| Normal | | 97 | (82.2%) | 7 | (58.3%) | 104 | (80.0%) |  |
| Angled | | 21 | (17.8%) | 5 | (41.7%) | 26 | (20.0%) |  |
| **Sinus rhythm** | |  |  |  |  |  |  | 0.004 |
| No | | 2 | (1.7%) | 2 | (16.7%) | 4 | (3.1%) |  |
| Yes | | 116 | (98.3%) | 10 | (83.3%) | 126 | (96.9%) |  |
| **NYHA class, N=129** | |  |  |  |  |  |  | <0.001 |
| 1 | | 103 | (88.0%) | 6 | (50.0%) | 109 | (84.5%) |  |
| 2 | | 13 | (11.1%) | 2 | (16.7%) | 15 | (11.6%) |  |
| 3 | | 1 | (0.9%) | 3 | (25.0%) | 4 | (3.1%) |  |
| 4 | | 0 | (0.0%) | 1 | (8.3%) | 1 | (0.8%) |  |

LV= left ventricle; RVEDV= right ventricle end-diastolic volume; RVESV= right ventricle end-systolic volume; RA= right atrium; IVS= interventricular septum; RVOT= right ventricular outflow tract; CMR= cardiac magnetic resonance; TOF= Tetralogy of Fallot; PA= pulmonary artery (arteries); PTA= percutaneous transluminal angioplasty; LVEF= left ventricle ejection fraction; RVEDVi= right ventricle end-diastolic volume indexed to BSA; RVESVi= right ventricle end-systolic volume indexed to BSA; MPA= main pulmonary artery; NYHA= New York Heart Association.

**Supplementary table 3**

CMR and clinical variables correlated to NYHA class increase.

| **Variable** | **No outcome (N=106)** | | | **Outcome (N=24)** | | **All patients (N=130)** | | **p** |
| --- | --- | --- | --- | --- | --- | --- | --- | --- |
| **VO2max pre-homograft (%), N=38** | | 73.0 | (66.0 -83.0) | 47.0 | (45.0 -51.0) | 72.0 | (62.0 -83.0) | 0.021 |
| **VO2max pre-homograft (ml/kg/min), N=38** | | 28.6 | (23.9 -31.7) | 16.9 | (13.6 -17.8) | 27.0 | (20.8 -31.0) | 0.002 |
| **Height (m)** | | 1.7 | (1.6 -1.7) | 1.6 | (1.6 -1.7) | 1.7 | (1.6 -1.7) | 0.007 |
| **LV EDV (ml)** | | 132.5 | (112.0 -158.8) | 112.9 | (100.1 -147.4) | 129.1 | (108.4 -156.3) | 0.028 |
| **LV ESV (ml)** | | 57.1 | (44.4 -68.6) | 50.0 | (39.1 -55.6) | 55.5 | (43.2 -66.4) | 0.036 |
| **RA longitudinal diameter (cm)** | | 5.0 | (4.2 -5.5) | 5.4 | (4.9 -6.3) | 5.0 | (4.4 -5.6) | 0.012 |
| **RVOT aneurysm coronal diameter (mm), N=32** | | 35.0 | (32.0 -41.0) | 51.0 | (42.0 -53.0) | 35.5 | (33.5 -42.5) | 0.016 |
| **Subsequent PPVI** | |  |  |  |  |  |  | 0.029 |
| No | | 105 | (99.1%) | 22 | (91.7%) | 127 | (97.7%) |  |
| Yes | | 1 | (0.9%) | 2 | (8.3%) | 3 | (2.3%) |  |
| **NYHA class, N=129** | |  |  |  |  |  |  | <0.001 |
| 1 | | 95 | (90.5%) | 14 | (58.3%) | 109 | (84.5%) |  |
| 2 | | 9 | (8.6%) | 6 | (25.0%) | 15 | (11.6%) |  |
| 3 | | 1 | (1.0%) | 3 | (12.5%) | 4 | (3.1%) |  |
| 4 | | 0 | (0.0%) | 1 | (4.2%) | 1 | (0.8%) |  |

RA= right atrium; RVOT= right ventricular outflow tract; NYHA= New York Heart Association.
